# Fatigue level changes with time in long-term Hodgkin and non-Hodgkin lymphoma survivors: a joint EORTC-LYSA cross-sectional study

**DOI:** 10.1186/s12955-019-1186-x

**Published:** 2019-07-02

**Authors:** Raphaël Busson, Marleen van der Kaaij, Nicolas Mounier, Berthe M. P. Aleman, Catherine Thiéblemont, Aspasia Stamatoullas, Vincent Ribrag, Hervé Tilly, Corinne Haioun, René-Olivier Casasnovas, Hanneke C. Kluin-Nelemans, Michel Henry-Amar

**Affiliations:** 1 École Doctorale MIIS, University of Caen-Normandie, 14032 Caen, France; 2 Centre de Traitement des Données du Cancéropôle Nord-Ouest, Plateforme de Recherche Clinique Ligue Contre le Cancer, Centre François Baclesse, 3 Avenue Général Harris, 14076 Caen, Cedex 5 France; 3 Department of Internal Medicine, Leiden University Medical Centre, Albinusdreef 2, 2333 ZA Leiden, the Netherlands; 4 Service d’Onco-hématologie, Université Côte d’Azur, Centre Hospitalier Universitaire de Nice, Hôpital l’Archet 2, 151 Route Saint-Antoine de Ginestière, BP 3079, 06202 Nice, Cedex 3 France; 5 Department of Radiotherapy, The Netherlands Cancer Institute, Plesmanlaan 121, 1066 CX Amsterdam, the Netherlands; 6 Service d’Hématologie, AP-HP CHU Saint-Louis, 1 Avenue Claude Vellefaux, 75010 Paris, France; 7 Service d’Hématologie, Centre Henri Becquerel, Rue d’Amiens, 76000 Rouen, France; 8 Service d’Hématologie, Gustave Roussy Cancer Campus, 114 Rue Edouard Vaillant, 94805 Villejuif, Cedex France; 9 Service d’Hématologie, AP-HP CHU Henri Mondor, 51 Avenue du Maréchal de Lattre de Tassigny, 94010 Créteil, France; 10 Service d’Hématologie, CHRU de Dijon Bourgogne, Hôpital Le Bocage, 2 Boulevard Maréchal de Lattre of Tassigny, 21000 Dijon, France; 11 Department of Haematology, University Medical Centre Groningen, University of Groningen, PO Box 30.001, 9700 RB Groningen, the Netherlands

**Keywords:** Hodgkin lymphoma, Non-Hodgkin lymphomas, Long-term survivors, Fatigue, Cross-sectional study

## Abstract

**Background:**

Long-term lymphoma survivors often complain of persistent fatigue that remains unexplained. While largely reported in Hodgkin lymphoma (HL), long-term fatigue is poorly documented in non-Hodgkin lymphomas (NHL). Data collected in two cohort studies were used to illustrate the fatigue level changes with time in the two populations.

**Methods:**

Two cross-sectional studies were conducted in 2009–2010 (HL) and in 2015 (NHL) in survivors enrolled in European Organisation for Research and Treatment of Cancer (EORTC) Lymphoma Group and Lymphoma Study Association (LYSA) trials. The same protocol and questionnaires were used in both studies including the Multidimensional Fatigue Inventory (MFI) tool to assess fatigue and a checklist of health disorders. Multivariate linear regression models were used in the two populations separately to assess the influence of time since diagnosis and primary treatment, age, gender, education level, cohabitation status, obesity and health disorders on fatigue level changes. Fatigue level changes were compared to general population data.

**Results:**

Overall, data of 2023 HL and 1619 NHL survivors with fatigue assessment available (99 and 97% of cases, respectively) were analyzed. Crude levels of fatigue were similar in the two populations. Individuals who reported health disorders (61% of HL and 64% of NHL) displayed higher levels of fatigue than those who did not (*P* <  0.001). HL survivors showed increasing fatigue level with age while in NHL survivors mean fatigue level remained constant until age 70 and increased beyond. HL survivors showed fatigue changes with age higher than those of the general population with health disorders while NHL survivors were in between those of the general population with and without health disorders.

**Conclusions:**

Among lymphoma survivors progressive increase of fatigue level with time since treatment completion is a distinctive feature of HL. Our data suggest that changes in fatigue level are unlikely to only depend on treatment complications and health disorders. Investigations should be undertaken to identify which factors including biologic mechanisms could explain why a substantial proportion of survivors develop high level of fatigue.

## Background

Among disease-related symptoms cancer patients generally complain of, fatigue is probably the most frequently reported [1]. Common during treatment, fatigue usually decreases with time to normal levels within few months or years after successful treatment [2, 3]. In up to one-third of patients fatigue can persist 10 years or more but studies reporting on fatigue in long-term cancer survivors are limited [4, 5]. Most of these studies concerned individuals who survived Hodgkin lymphoma (HL), few non-Hodgkin lymphomas (NHL) or both ([2, 3, 6–13], reviews in [14, 15]).

Survival improvement in HL brought physicians’ attention to persistent fatigue that was observed in a substantial proportion of survivors, including those who survived childhood HL, which might exceed 65% [2, 16]. In two series of lymphoma survivors enrolled in the European Organisation for Research and Treatment of Cancer (EORTC) Lymphoma Group and the Lymphoma Study Association (LYSA) clinical trials, the proportions of individuals who reported long-term fatigue were 64 and 62% in HL and NHL survivors, respectively [2, 17]. Factors generally associated with increased prevalence of fatigue or increased fatigue level were age, female gender, low education level, and presence of health disorders. In contrast, persistent fatigue was unrelated to primary treatment intensity and treatment given at relapse [14].

Fatigue assessment often varies between studies (longitudinal or cross-sectional) both in time since treatment end and questionnaires used. Validated specific questionnaires mostly used were: the Fatigue Questionnaire (FQ) [18] used in 15 HL and three NHL studies; the Functional Assessment of Chronic Illness Therapy-Fatigue (FACIT-F) [19] used in four HL studies; the Multidimensional Fatigue Inventory (MFI) [20] used in five HL and one NHL studies; and the Fatigue Assessment Scale (FAS) [21] used in three HL studies. Among validated general questionnaires that include symptoms items on fatigue, the most often used was the EORTC Quality-of-Life Core Questionnaire (QLQ-C30) [22] mentioned in 15 HL and two NHL studies.

The heterogeneity of fatigue assessment tools used, the patients’ characteristics collected including health disorders and the study designs preclude any reliable comparisons and conclusions on whether prevalence of persistent fatigue differs within survivors of lymphomas or between cancer survivors. We had the opportunity to analyze fatigue level changes in two cohorts of long-term survivors of HL and NHL based on the same study design and instruments with focus on the effect of age and follow-up.

## Patients and methods

### Study design

In 2009–2010 the EORTC Lymphoma Group and the LYSA have designed a cross-sectional study to collect information on socio-demographic characteristics, health situation and fatigue of HL survivors enrolled in the nine clinical trials that were conducted from 1964 to 2004. Two self-administered questionnaires were used in addition to clinical data prospectively collected and stored in a unique secured database at the EORTC Head Quarter in Brussels, Belgium. In 2015 the LYSA repeated the cross-sectional study in NHL survivors enrolled in the 12 clinical studies that were conducted from 1993 to 2010. The same two self-administered questionnaires were used in addition to clinical data prospectively collected and stored in a unique secured database at the LYSA Academic Research Organisation, Centre Hospitalier Lyon-Sud, Pierre-Bénite, France. Survivors were eligible if they had no active lymphoma, had follow-up of 5 years or more, and were free from any cancer treatments since 4 years. Detailed descriptions of the cross-sectional studies were previously published [23, 24].

### Ethics approval and consent to participate

Authorizations were obtained from the EORTC Scientific and Ethical Committees, the ethical committee and legal authorities in France, and local ethical committees at each participating hospital in other European countries. The study was performed in accordance with the Declaration of Helsinki.

Survivors voluntarily participated in the survey and signed informed consent.

### Questionnaires and data collection

The Life Situation Questionnaire (LSQ) addresses issues not available in other validated questionnaires including: socio-demographic data, cohabitation status and highest level of education [25]; parenthood data; education, work, and insurance; health situation including height, weight, and detailed information (checklist) on post-treatment health disorders and current treatments; and social situation [23]. Self-reported health disorders that had occurred after the end of the lymphoma treatment were grouped as follows: cardiovascular, pulmonary, and musculoskeletal disorders; severe infections; anxiety; depression; and history of second cancer. No attempt was a posteriori made to confirm these diseases using data available in either medical records or computerized clinical data.

The MFI questionnaire was used to address the topic of fatigue [20]. It consisted of 20 items, each item coded 1 to 5. From the 20 items, five scales were generated: general fatigue, physical fatigue, reduced activity, reduced motivation, and mental fatigue. Each scale was constructed by summation of its four items; the total obtained was transformed to a linear score ranging from 0 to 100. Zero indicated absence of fatigue and the higher the score, the higher the level of fatigue.

Baseline patient characteristics and treatments administered were retrieved from the clinical databases. Age at survey was obtained by subtracting the date of birth to the date the questionnaires were completed. Follow-up time was obtained by subtracting the date of randomization or the date of first treatment to the date the questionnaires were completed. The weight (kg)-to-height (m^2^) ratio was used to calculate the body mass index (BMI) at the time of survivorship assessment; obesity was defined as a BMI ≥ 30 kg/m^2^.

### Population study

**Fig. 1:**
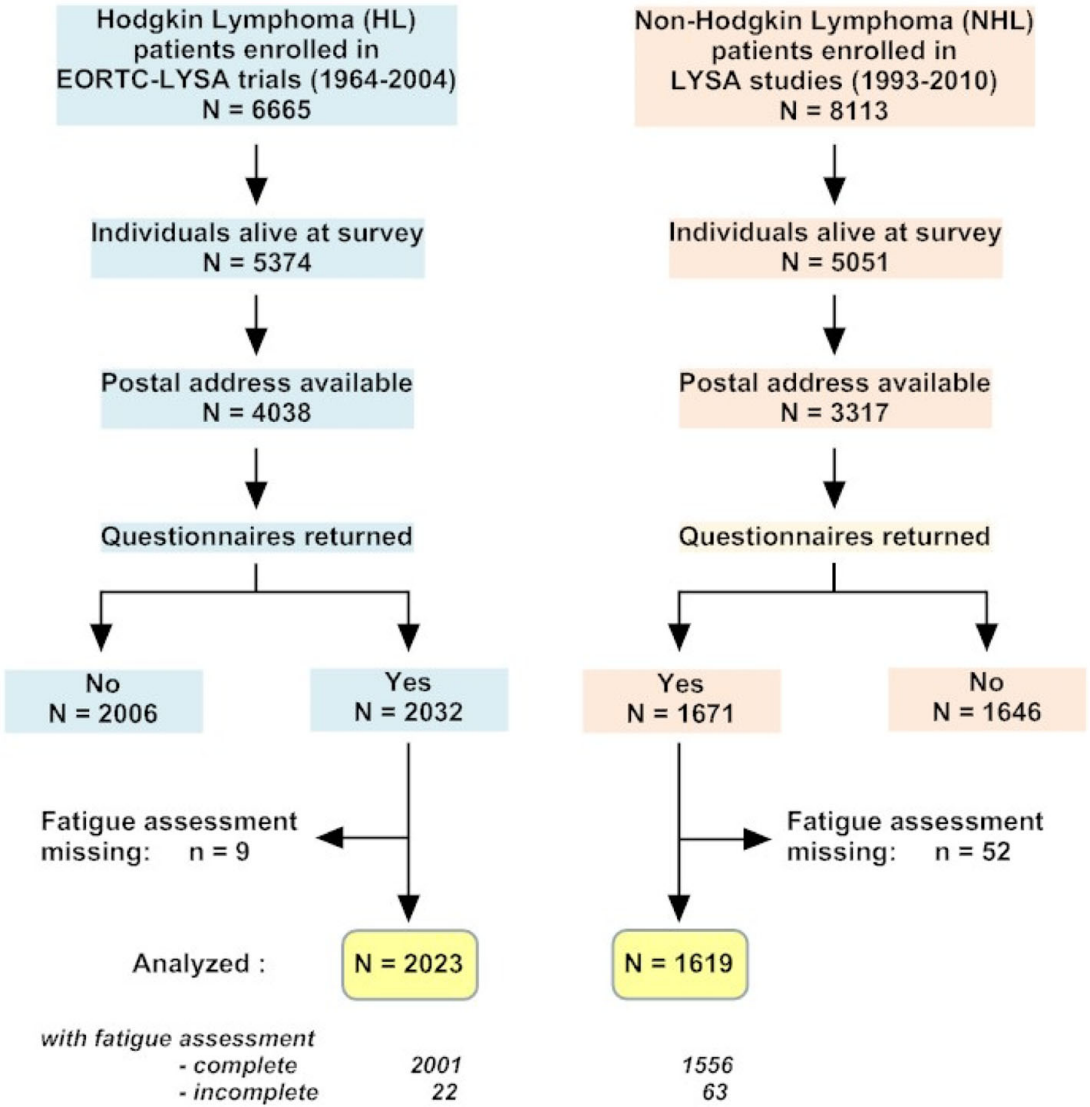
Study profile and enrollment

Overall, 6665 and 8113 patients were enrolled in the HL and NHL clinical studies, respectively. Of these, 5374 HL (80.6%) and 5051 NHL (62.3%) patients were alive at the time the surveys started. A postal address was obtained for 4038 HL and 3317 NHL individuals. Of these half participated in the survey giving 2032 HL (50.3%) and 1671 NHL (50.4%) cases available for analysis (Fig. 1).

### Statistical analysis

Clinical characteristics, treatment protocols, and clinical outcome of NHL survivors have been recently published [17]; those of HL survivors are under submission for publication in another journal. Demographic characteristics, follow-up time since treatment initiation and medical history as reported by survivors were described using numbers and proportions for HL and NHL separately. Because incidence and initial clinical characteristics and treatment protocols differ between HL and NHL patients, no comparisons were made between the two populations. Fatigue scores at the time of survivorship assessment were first expressed using crude mean and standard deviation for the five dimensions of the MFI assessment tool for HL and NHL separately. Adjusted mean fatigue scores were also estimated using linear regression models with gender and education level, age, cohabitation status and obesity at time of survivorship assessment as covariates. The impact of self-reported health disorders on fatigue level was analyzed within each lymphoma population using adjusted t-test. Statistical tests were two-sided with statistical significance defined as a *P* <  0.05.

Analysis of fatigue level changes in long-term survivors of HL and NHL was performed on cases with fatigue assessment (at least one dimension score) available. Multivariate linear regression models were used to assess the influence of age and time since diagnosis and treatment as covariates on changes of the five fatigue level scores, i.e. general fatigue, physical fatigue, mental fatigue, reduced activity, and reduced motivation. Variables included in the models were age at the time of survivorship assessment, follow-up time, gender, education level, cohabitation status, obesity and presence of health disorders at fatigue assessment. Primary treatments (including autologous stem-cell transplantation administered upfront in NHL patients) were not included in the models because they did not influence long-term fatigue levels as previously shown [2, 3, 14, 17]. Salvage treatments delivered for a relapse were not considered as well for the same reason. In the results, the intercept (reference score) is the estimation of the mean fatigue score for a male aged 20 years, living with partner, with follow-up time equals zero. For age and follow-up time since treatment initiation the regression coefficient β estimates the change in score associated with a 10-year increase. Cases aged ≥70 years (10% of the population) were grouped because in a previous study focusing on NHL it was shown that fatigue level remained unchanged until 69 years of age and significantly increased beyond [17]. A score can be estimated by simply adding the following terms: intercept + (β_age < 70_ x (age-20)/10) + β_age ≥ 70_ + (β_follow-up_ x follow-up time/10) + β_i_V_i_ (where V_i_ represents any covariate included in the model and β_i_ its regression coefficient). Predicted fatigue scores were plotted according to age assuming that cases had been treated at age 45 years. No data being available in lymphoma survivors on minimal (clinical) important difference of fatigue changes based on the MFI, the regression coefficient β estimates were used to test for slopes different from zero.

An attempt was made to compare fatigue level changes with time to general population data in which fatigue level was assessed by use of the MFI instrument [26]. Data consisted of a sample of 1082 individuals (50.3% women; age range, 20 to 79) with equal size aged strata for whom socio-demographic determinants were available such as: education level, cohabitation status, and presence of self-reported health disorders (i.e. somatic or psychological disorders including cancer). Estimations of fatigue levels by age were made with adjustment on gender, education level and cohabitation status. Fatigue levels by age were plotted for individuals without and with health disorders separately. No statistical comparisons were made.

Data were analyzed at the *Centre de Traitement des Données du Cancéropôle Nord-Ouest*, *Plateforme de Recherche Clinique Ligue Contre le Cancer,* Centre François Baclesse (Caen, France). All analyses were performed with STATA software (version 14.2; STATA Corp, College Station, Texas 77,845 USA).

## Results

Of the 2032 HL survivors and the 1671 NHL survivors who returned the LSQ and the fatigue assessment questionnaires, 2023 (99%) HL cases and 1619 (98%) NHL cases had fatigue assessment available and were included in the present analysis (Fig. 1). Among HL cases, 197 cases had radiation therapy alone, 345 were given chemotherapy alone, and 1447 received combined therapy as part of their primary treatment; the treatment was not specified in 34 cases. Among NHL cases (1135 with diffuse large B-cell lymphoma, 461 with follicular lymphoma, and 23 with T-cell lymphoma), primary treatment consisted of conventional chemotherapy in 780 cases, intensive chemotherapy (mainly high-dose cyclophosphamide, doxorubicin, vincristine, prednisone [CHOP] or CHOP-like such as adriamycin, cyclophosphamide, vindesine, bleomycin, prednisone [ACVBP]) alone in 505, or combined with autologous stem cell transplantation in 334 [17]. Rituximab was administered to 807 cases.

### HL and NHL characteristics

As expected, NHL cases were 15 years older than HL cases in average (Table 1). The age also explained the excess of low educated (elementary school) cases and the higher proportion of cases living without partner in NHL population. The number of health disorders reported by the participants at the time of survivorship assessment were similarly distributed in HL and NHL cases, i.e. 61.5 and 64.4%, respectively; those reporting three or more health disorders were 26.0 and 26.9%. However, NHL cases reported twice as many history of second cancer than HL cases. At the time of survivorship assessment HL and NHL cases expressed similar crude levels of fatigue than NHL cases in all dimensions. Levels of fatigue adjusted on gender, age, education level, cohabitation status and obesity were influenced by the presence of health disorders at the time the survivorship assessment was made. HL and NHL survivors reporting health disorders (any types) had significantly higher levels of fatigue than those who did not report health disorders (*P* <  0.001) (Table 2).

**Table 1:** Lymphoma survivors’ characteristics at the time of survivorship assessment

	Hodgkin lymphoma *N* = 2023		Non-Hodgkin lymphomas *N* = 1619	
	N	(%)	N	(%)
Age				
Mean (sd)	47.8	(12.3)	62.9	(12.7)
Median (min - max)	46.8	(24–85)	63.9	(24–92)
20–39 years	608	(30.1)	82	(5.1)
40–49 years	573	(28.3)	189	(11.7)
50–49 years	482	(23.8)	345	(21.3)
60–69 years	268	(13.3)	548	(33.8)
70–79 years	77	(3.8)	310	(19.1)
≥ 80 years	15	(0.7)	145	(9.0)
Gender				
Male	994	(49.1)	882	(54.5)
Female	1029	(50.9)	737	(45.5)
Education level				
University	736	(37.2)	516	(33.6)
High school	646	(32.6)	480	(31.2)
College	479	(24.2)	282	(18.3)
Elementary school	119	(6.0)	260	(16.9)
Unspecified	43		81	
Years since treatment start, mean (sd)	15.7	(7.6)	12.8	(4.5)
5 to 9 years	423	(20.9)	511	(31.6)
10 to 14 years	665	(32.9)	561	(34.6)
15 to 19 years	469	(23.2)	362	(21.4)
≥ 20 years	466	(23.0)	185	(11.4)
Cohabitation status				
Living without partner				
Yes	398	(19.7)	436	(26.9)
No	1625	(80.3)	1183	(73.1)
Body Mass Index (BMI)				
BMI ≥ 30 kg/m^2^	231	(11.4)	264	(16.3)
BMI < 30 kg/m^2^	1792	(88.6)	1355	(83.7)
Self-reported health disorders				
Cardiovascular disorders ^a^				
Yes	442	(21.9)	327	(20.2)
No	1581	(78.1)	1292	(79.8)
Pulmonary disorders ^a^				
Yes	164	(8.1)	108	(6.7)
No	1859	(91.9)	1511	(93.3)
Severe infections ^a^				
Yes	288	(14.2)	200	(12.4)
No	1735	(85.8)	1419	(87.6)
Musculoskeletal disorders ^a^				
Yes	172	(8.5)	142	(8.8)
No	1851	(91.5)	1477	(91.2)
Anxiety				
Yes	184	(9.1)	216	(13.3)
No	1839	(90.9)	1403	(86.7)
Depression or suicide attempt				
Yes	251	(12.4)	153	(9.5)
No	1772	(87.6)	1466	(90.5)
Number of self-reported health disorders excluding second cancers				
0	780	(38.5)	577	(35.6)
1	423	(20.9)	382	(23.6)
2	295	(14.6)	225	(13.9)
3	202	(10.0)	162	(10.0)
≥ 4	323	(16.0)	273	(16.9)
History of second cancer				
Yes	61	(3.0)	127	(7.8)
No	1962	(97.0)	1492	(92.2)
Fatigue assessment				
MFI scores, crude mean (sd ^b^)				
General fatigue (2020/1605) ^c^	44.8	(29.4)	42.5	(26.4)
Physical fatigue (2018/1577)	38.6	(29.8)	36.7	(27.3)
Reduced activity (2017/1587)	31.8	(26.4)	33.4	(23.9)
Reduced motivation (2018/1585)	26.9	(24.4)	27.2	(23.4)
Mental fatigue (2007/1586)	31.4	(27.4)	28.3	(25.2)

^a^Cardiovascular disorders: heart valve problem, heart rhythm disorder, heart failure, peripheral artery disease, stroke, thrombosis

Pulmonary disorders: pleurisy, lung function deterioration, chronic obstructive pulmonary disease

Severe infections: zona infection, herpes zoster, hepatitis B, hepatitis C, tuberculosis

Musculoskeletal disorders: avascular necrosis of bone, muscular fibrosis, severe osteoarthritis

^b^sd indicates standard deviation

^c^Number of Hodgkin and non-Hodgkin lymphoma survivors with fatigue assessment available

**Table 2:** Adjusted mean MFI scale scores by presence of health disorders at survivorship assessment

	Lymphoma type	Somatic or psychological diseases						Adjusted *P-value*
		Absence			Presence			
		No	Adjusted mean ^a^	(95% CL) ^b^	No	Adjusted mean	(95% CL)	
MFI scores								
General fatigue	HL	780	35.6	(33.7;37.6)	1240	50.5	(49.0;52.1)	< 0.001
	NHL	573	32.5	(30.4;34.5)	1032	48.0	(46.5;49.5)	< 0.001
Physical fatigue	HL	777	29.1	(27.2;31.1)	1241	44.6	(43.0;46.1)	< 0.001
	NHL	567	26.8	(24.6;28.9)	1010	42.3	(40.7;43.9)	< 0.001
Reduced activity	HL	779	25.9	(24.2;27.7)	1238	35.4	(34.0;36.8)	< 0.001
	NHL	573	27.7	(25.9;29.6)	1014	36.6	(35.2;38.0)	< 0.001
Reduced motivation	HL	779	22.8	(21.1;24.4)	1239	29.4	(28.1;30.7)	< 0.001
	NHL	571	21.9	(20.1;23.7)	1014	30.3	(28.9;31.6)	< 0.001
Mental fatigue	HL	774	24.9	(23.0;26.8)	1233	35.4	(33.9;36.9)	< 0.001
	NHL	570	21.7	(19.7;23.7)	1016	31.9	(30.4;33.4)	< 0.001

*HL* Hodgkin lymphoma, *NHL* non-Hodgkin lymphomas

^a^Adjustment using linear regression model with gender and education level, and age, cohabitation status and obesity at fatigue assessment as covariates

^b^95% CL indicates 95% confidence limits of adjusted mean score estimation

### Fatigue level changes with age and follow-up time

**Fig. 2:**
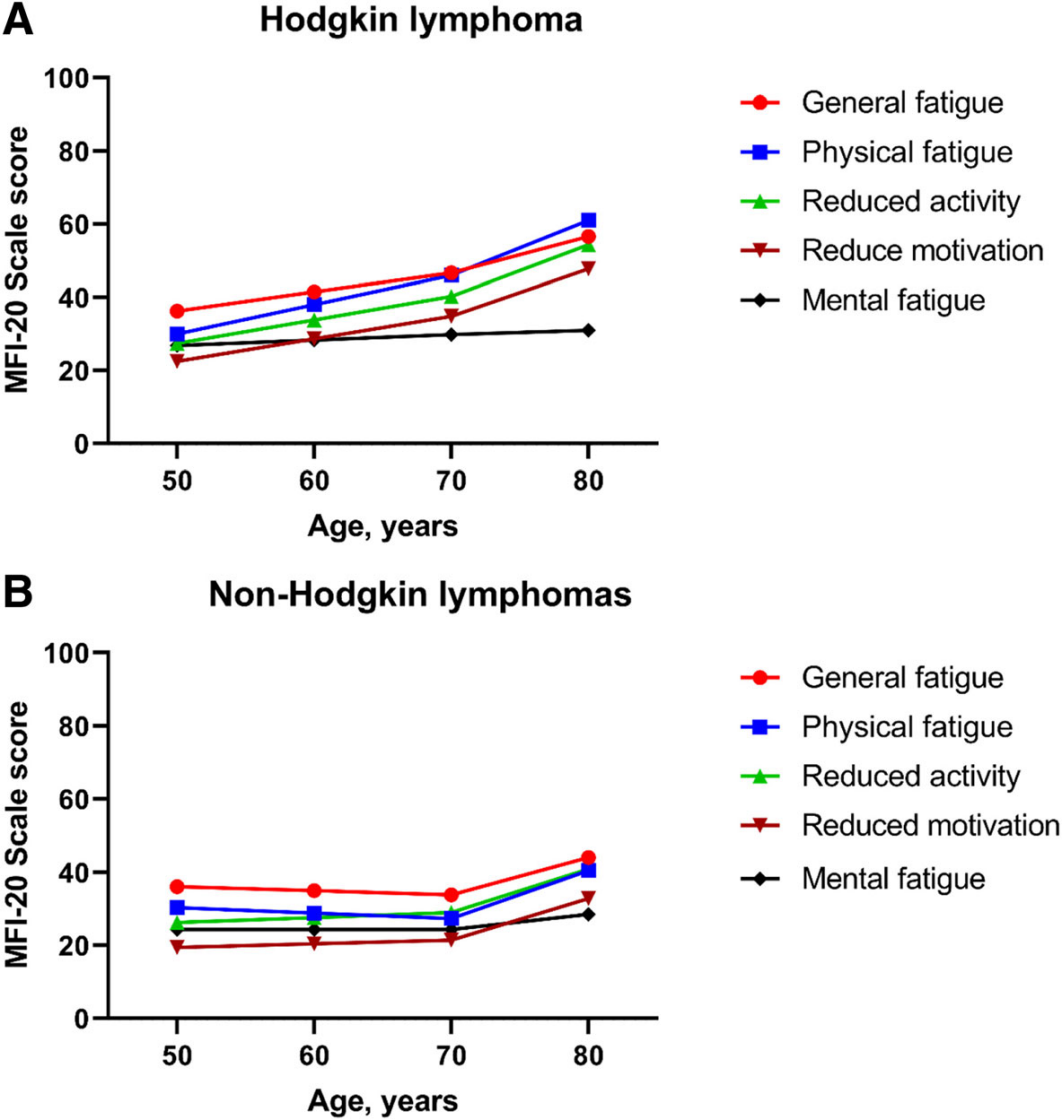
MFI assessment: Changes of mean fatigue scores in HL (panel **a**) and NHL (panel **b**) with age. Predicted mean fatigue scores for high educated, non-obese male survivors living with partner, treated at 45 years of age. Curves start at age 50 because all survivors had at least 5 years of follow-up at the time of survivorship assessment. On the X-axis, age minus 45 equals follow-up

The effects of age and time since treatment (adjusted on gender, age, education level, cohabitation status, obesity and the presence of health disorders) on the five dimensions of fatigue are shown in Table 3. In HL, mean fatigue levels significantly increased from age 20 to 69 for all dimensions except mental fatigue. In individuals aged 70 or older, age increased the mean fatigue levels for physical fatigue, reduced activity and reduced motivation only. Similarly, mean fatigue levels increased with follow-up time: a marked influence was noticed for physical fatigue; it was less important for general fatigue, reduced activity and reduced motivation. In NHL, an increase in mean fatigue levels with increased age from 20 to 69 years was observed for reduced activity. In contrast, physical fatigue level decreased with increasing age until 69 years. In older cases, the effect of age was of the same magnitude in all dimensions except mental fatigue. However, in contrast to HL, follow-up time did not influence fatigue. The combined influence of age and follow-up time on mean fatigue scores are illustrated in Fig. 2. The figures show the predicted mean fatigue scores 5 years and beyond the start of primary treatment for non-obese highly educated male survivors treated at 45 years of age, and living with partner. Main differences between HL and NHL are seen before 70 years of age with fatigue increasing in HL (Fig. 2a) and being stable or decreasing in NHL (Fig. 2b). For example, the negative effect of increasing age (β = − 1.2, Table 3) on general fatigue was more pronounced by follow-up time (β = − 1.3) ending at a slight decrease of fatigue score with increasing age in NHL. Beyond 70 years of age, the curves paralleled whatever the fatigue dimension. The same analyses were repeated on the subgroup of survivors who never relapsed of their disease, and who had a HL, a diffuse large B-cell lymphoma, or a follicular lymphoma. Of the 3642 cases, 514 (14.1%) were excluded either because they experienced a relapse (205 HL and 290 NHL), or were of T-cells histological type (*n* = 19). Overall, results remained unchanged.

**Table 3:** Multiple linear regression models on long-term fatigue using MFI assessment

			Hodgkin lymphoma		Non-Hodgkin lymphomas	
			Coef β (sd)	*P-value*	Coef β (sd)	*P-value*
General fatigue	Reference score (intercept)		23.7 (1.9)	*< 0.001*	32.0 (2.9)	*< 0.001*
	Age at time of fatigue assessment					
	per 10-yr increase	20–69 yrs	1.3 (0.6)	*0.047*	−1.2 (0.7)	*0.089*
		≥ 70 yrs	5.0 (5.1)	*0.328*	9.5 (1.7)	*< 0.001*
	Years since treatment start					
	per 10-yr increase		1.9 (0.8)	*0.018*	−1.3 (1.0)	*0.194*
	Gender	Male	0.0		0.0	
		Female	7.1 (1.2)	*< 0.001*	5.2 (1.3)	*< 0.001*
	Education level ^a^	High	0.0		0.0	
		Low	3.5 (1.4)	*0.015*	1.6 (1.4)	*0.240*
	Cohabitation status					
	living with partner	Yes	0.0		0.0	
		No	4.7 (1.6)	*0.003*	1.4 (1.4)	*0.322*
	Obesity ^b^	No	0.0		0.0	
		Yes	7.6 (2.0)	*< 0.001*	5.8 (1.7)	*0.001*
	Health disorders	No	0.0		0.0	
		Yes	14.6 (1.3)	*< 0.001*	15.6 (1.3)	*< 0.001*
Physical Fatigue	Reference score (intercept)		13.3 (1.9)	*< 0.001*	27.4 (3.0)	*< 0.001*
	Age at time of fatigue assessment					
	per 10-yr increase	20–69 yrs	2.3 (0.6)	*0.001*	−1.6 (0.7)	*0.033*
		≥ 70 yrs	10.1 (5.0)	*0.045*	12.2 (1.7)	*< 0.001*
	Years since treatment start					
	per 10-yr increase		3.7 (0.8)	*< 0.001*	−1.0 (1.0)	*0.328*
	Gender	Male	0.0		0.0	
		Female	5.2 (1.2)	*< 0.001*	2.8 (1.3)	*0.034*
	Education level ^a^	High	0.0		0.0	
		Low	3.5 (1.4)	*0.012*	2.6 (1.4)	*0.074*
	Cohabitation status					
	living with partner	Yes	0.0		0.0	
		No	4.8 (1.6)	*0.002*	2.2 (1.5)	*0.150*
	Obesity ^b^	No	0.0		0.0	
		Yes	10.3 (1.9)	*< 0.001*	6.9 (1.8)	*< 0.001*
	Health disorders	No	0.0		0.0	
		Yes	14.7 (1.3)	*< 0.001*	15.6 (3.0)	*< 0.001*
Reduced activity	Reference score (intercept)		11.4 (1.7)	*< 0.001*	17.3 (2.6)	*< 0.001*
	Age at time of fatigue assessment					
	per 10-yr increase	20–69 yrs	3.4 (0.6)	*< 0.001*	1.6 (0.6)	*0.011*
		≥ 70 yrs	12.8 (4.6)	*0.005*	11.3 (1.5)	*< 0.001*
	Years since treatment start					
	per 10-yr increase		1.6 (0.7)	*0.025*	−1.0 (0.9)	*0.273*
	Gender	Male	0.0		0.0	
		Female	−0.4 (1.1)	*0.723*	−0.6 (1.2)	*0.611*
	Education level ^a^	High	0.0		0.0	
		Low	4.2 (1.3)	*0.001*	2.2 (1.3)	*0.074*
	Cohabitation status					
	living with partner	Yes	0.0		0.0	
		No	7.9 (1.4)	*< 0.001*	3.2 (1.3)	*0.015*
	Obesity ^b^	No	0.0		0.0	
		Yes	5.6 (1.7)	*0.001*	5.2 (1.5)	*0.001*
	Health disorders	No	0.0		0.0	
		Yes	9.2 (1.2)	*< 0.001*	8.9 (1.2)	*< 0.001*
Reduced motivation	Reference score (intercept)		7.4 (1.6)	*< 0.001*	12.6 (2.6)	*< 0.001*
	Age at time of fatigue assessment					
	per 10-yr increase	20–69 yrs	3.7 (0.5)	*< 0.001*	0.8 (0.6)	*0.212*
		≥ 70 yrs	12.7 (4.2)	*0.003*	10.3 (1.5)	*< 0.001*
	Years since treatment start					
	per 10-yr increase		1.5 (0.7)	*0.022*	−0.5 (0.9)	*0.582*
	Gender	Male	0.0		0.0	
		Female	0.4 (1.0)	*0.692*	2.6 (1.1)	*0.020*
	Education level ^a^	High	0.0		0.0	
		Low	4.0 (1.2)	*0.001*	3.4 (1.2)	*0.005*
	Cohabitation status					
	living with partner	Yes	0.0		0.0	
		No	6.0 (1.3)	*< 0.001*	4.7 (1.3)	*< 0.001*
	Obesity ^b^	No	0.0		0.0	
		Yes	7.1 (1.6)	*< 0.001*	4.0 (1.5)	*0.008*
	Health disorders	No	0.0		0.0	
		Yes	6.5 (1.1)	*< 0.001*	8.4 (1.2)	*< 0.001*
Mental fatigue	Reference score (intercept)		19.3 (1.8)	*< 0.001*	21.4 (2.9)	*0.002*
	Age at time of fatigue assessment					
	per 10-yr increase	20–69 yrs	0.6 (0.6)	*0.318*	−0.9 (0.7)	*0.211*
		≥ 70 yrs	−0.2 (4.9)	*0.965*	3.4 (1.7)	*0.043*
	Years since treatment start					
	per 10-yr increase		−0.6 (0.8)	*0.431*	−0.2 (1.0)	*0.866*
	Gender	Male	0.0		0.0	
		Female	3.4 (1.2)	*0.005*	1.4 (1.3)	*0.282*
	Education level ^a^	High	0.0		0.0	
		Low	4.7 (1.4)	*0.001*	4.2 (1.4)	*0.002*
	Cohabitation status					
	living with partner	Yes	0.0		0.0	
		No	4.4 (1.5)	*0.003*	4.0 (1.4)	*0.006*
	Obesity ^b^	No	0.0		0.0	
		Yes	2.6 (1.9)	*0.159*	−0.1 (1.7)	*0.929*
	Health disorders	No	0.0		0.0	
		Yes	10.7 (1.3)	*< 0.001*	10.2 (1.3)	*< 0.001*

sd indicates standard deviation

^a^High education level indicates university or high school; low education level indicates college, elementary school or level unspecified

^b^Obesity indicates Body Mass Index (BMI) ≥ 30 kg/m^2^

### General population comparisons

**Fig. 3:**
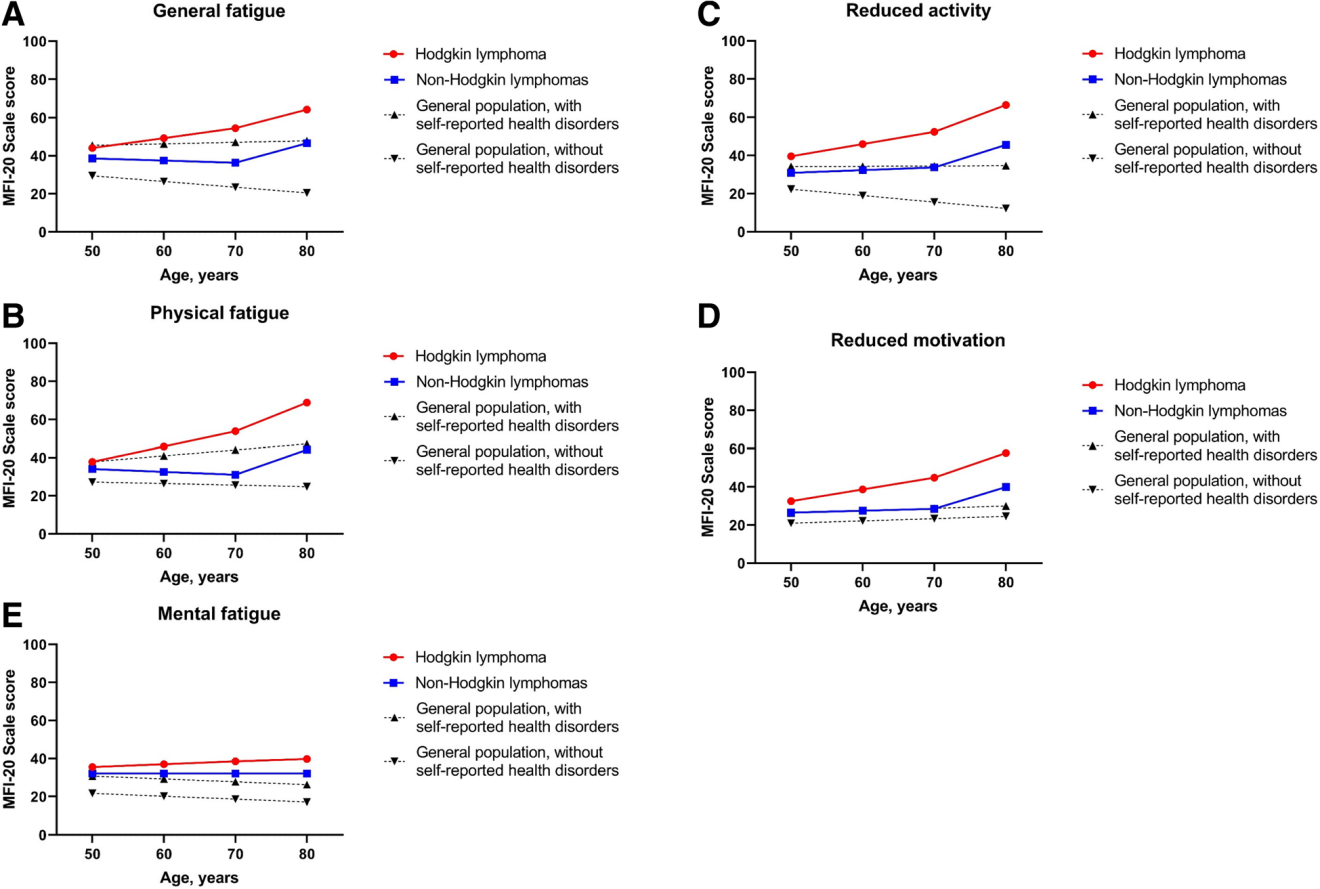
MFI assessment: Changes of mean fatigue scores in HL, NHL, and general population with age: General fatigue (panel **a**), physical fatigue (panel **b**), reduced activity (panel **c**), reduced motivation (panel **d**), and mental fatigue (panel **e**). Predicted mean fatigue scores (solid lines) for Hodgkin () and non-Hodgkin lymphomas () low educated males survivors, living alone, treated at 45 years of age. Predicted mean fatigue scores for general population (dash lines) with (▲) and without (▼) health disorders [17]

Predicted mean fatigue scores by age were higher for both HL and NHL survivors compared with general population data [26]. Illustrations are given for low educated males living without partner for whom those who survived HL had higher fatigue levels in all dimensions than individuals who survived NHL (Fig. 3a to e). For all scale scores, HL survivors (61.5% with health disorders) displayed changes with age higher than those of the general population with health disorders; in contrast plots for NHL survivors (64.4% with health disorders) were in between those of the general population with and without health disorders except for mental fatigue (Fig. 3e).

## Discussion

In the present paper, we report on fatigue changes with time in long-term survivors of lymphomas, an issue generally poorly documented concerning its quantitative aspect and particularly its relationships with health disorders. While persistent fatigue in HL survivors has brought interest of researchers since 1996 [27], the first publication focusing on NHL survivors was published in 2015 [7]. Above all, when comparisons are made between series of a given cancer localization or between cancer localizations, methodologies and instruments for fatigue assessment used often differ. In recent studies focusing on lymphomas, data from HL and NHL survivors were pooled when reporting on long-term fatigue [9–12]. We had the opportunity to develop two cross-sectional surveys with the aim to evaluate rehabilitation, health status, and long-term fatigue in survivors of lymphomas who participated in clinical protocols conducted by two European cooperative groups. In the two surveys, the same methodology and the same self-administered questionnaires were used [23, 24].

With only lymphoma survivors involved in the survey, our study shows that HL and NHL survivors display similar long-term fatigue levels in the five dimensions of the MFI assessment tool. Having or not health disorders does not change the conclusion. Changes of fatigue level can be modelled according to time since lymphoma treatment. Until age 69 years, except for mental fatigue, fatigue levels progressively increase with time in HL survivors. In NHL survivors, fatigue levels stay almost unchanged in all dimensions but two: for reduced activity a slow increase is observed; for physical activity a slow decrease is observed. Beyond 70 years of age, fatigue levels show parallel increases in both HL and NHL survivors, with HL figures always above that of NHL in all dimensions except mental fatigue.

In a cross-sectional study conducted in the general population the MFI questionnaire was used to assess the level of fatigue and a checklist was proposed to report health disorders supplemented by an open question about any other diseases [26]. In this sample, 39.7% of cases reported health disorders. Mean fatigue levels were higher (7 to 21 point difference depending of scale scores) in individuals with health disorders compared with those without health disorders. Overall, HL and NHL survivors have fatigue levels of the same magnitude than what is observed in general population cases with health disorders. HL survivors with or without self-reported health disorders always display higher levels of fatigue than general population cases with similar socio-demographic characteristics. In contrast, the figures differ in NHL survivors. Those with no self-reported health disorders have higher levels of general fatigue and reduced activity than individuals of the general population with the same characteristics. However, NHL survivors who report health disorders have levels of fatigue comparable to that of the general population with health disorders except for mental fatigue for which the levels are slightly increased. Using multiple regression analysis on general population data, age, gender, low education level, living without partner and presence of health disorders (mostly depression) significantly increased the level of fatigue with various impact according to scale scores [26]. These results were used to illustrate changes in fatigue levels according to age in HL, NHL, and general population with and without health disorders separately. The figures confirm that HL survivors suffer from long-term fatigue of similar magnitude if not higher than individuals with health disorders in the general population.

Our study confirms that a substantial proportion of long-term lymphoma survivors develop diseases that can favor the development or the persistence of fatigue. Although the numbers of individuals who complain of health disorders are rather similar among HL and NHL survivors, their types differ and we have shown that each of them have similar impact on the levels of fatigue [17, unpublished data]. Besides diseases, other individual characteristics can play a role on the development of fatigue such as a low education level, living without partner, and obesity. In contrast, the fatigue level is in almost all studies independent of treatments (primary treatment or given at relapse) as previously reported [2, 3, 14, 28]. It is also independent of NHL histological type-related treatments [7, 29].

It is unlikely that differences observed between HL and NHL survivors in changes of fatigue levels with age before 70 years can simply be explained by the presence of health disorders. Epstein-Barr virus (EBV) infection has long been described in classical HL and, in European countries, its prevalence ranges from 31 to 40% [30]. It is associated with increased cytokine levels [31]; and genome-level mutations responsible for cytokine production induce increased fatigue level in breast cancer survivors [32, 33]. Recently, a study performed in fatigued patients with solid tumors showed that a high level of IL-1 and IL-1 Ra cytokines correlates with high levels of fatigue [34]. Variations in neurotransmitter genes have also been associated with the development of chronic fatigue in breast cancer [35]. These results suggest that fatigue could have in part a genetic origin. On the other hand, a substantial proportion of newly diagnosed patients with HL display T-lymphocytopenia that can persist long after the disease is cured suggesting chronic immunologic impairment that can relate to genetic or environmental origin [36]. NHL survivors might also suffer from immunodeficiency as indirectly suggested by a history of infections prior to diagnosis [37]. However, no genetic studies focusing on immunodeficiency and fatigue in lymphoma patients, at diagnosis or long after the treatment was completed, have been conducted so far. HL patients can also present at diagnosis with lymphocyte telomeres length shorter than that of healthy individuals [38]. Since leukocyte telomeres length reduction was shown to be associated with fatigue level in nondisabled older adults [39], one can question whether the association of multiple genetic mutations pre-existing the disease could concur to pre-treatment and/or long-term abnormal fatigue in lymphoma patients.

## Conclusions

Persistent fatigue is a symptom commonly reported by cancer survivors [1] interfering with patients’ (quality of) life. Often studied in Hodgkin lymphoma, its prevalence is poorly known in non-Hodgkin lymphomas. We used self-reported fatigue data from two European cross-sectional studies conducted in long-term survivors. In both studies fatigue level was assessed and health disorders were collected using the same questionnaires. Overall, 2023 and 1619 individuals with Hodgkin and non-Hodgkin lymphomas were available allowing comparisons of fatigue level changes with time based on multivariate linear regression modeling. At time of survivorship assessment, Hodgkin and non-Hodgkin lymphoma survivors expressed similar crude mean levels of fatigue in all MFI dimensions. In both groups, fatigue levels were linked to the presence of health disorders (*P* <  0.001). In Hodgkin lymphoma survivors, fatigue levels increased linearly with age; in non-Hodgkin lymphoma survivors, fatigue levels remained constant until age 70 and increased afterwards parallel to what was observed in Hodgkin lymphoma. Compared to general population data, Hodgkin lymphoma survivors showed fatigue level changes with age parallel and higher than those of the general population with health disorders. In contrast, non-Hodgkin lymphoma survivors displayed fatigue level changes with age in between those of the general population with and without health disorders.

Our study is the first reporting on direct comparison between Hodgkin and non-Hodgkin lymphoma survivors. It also provides indications on fatigue level changes with time with indirect comparison with general population data. No medical explanations exist for why fatigue develops or persists in some patients. In particular, long-term fatigue is unrelated to lymphoma treatments [2, 3, 14, 17]. Therefore, time has probably come to investigate its biologic origin. Conclusive results could then be used to select patients who would benefit from various tertiary prevention interventions to manage or prevent the development of persistent fatigue [40–42].

## Data Availability

R Busson, M Henry-Amar and N Mounier had full access to the data which is stored in a secured database at the Centre Hospitalo-Universitaire, Hôpital l’Archet, Nice, France (non-Hodgkin lymphoma data) and at the EORTC Head Quarter in Brussels, Belgium (Hodgkin lymphoma data). The datasets used and analyzed during the current study are available from the corresponding author on reasonable request.

## Abbreviations

ACVBP: Adriamycin, cyclophosphamide, vindesine, bleomycin, prednisone
BMI: Body mass index
CHOP: Cyclophosphamide, doxorubicin, vincristine, prednisone
EBV: Epstein-Barr virus
EORTC: European Organisation for Research and Treatment of Cancer
HL: Hodgkin lymphoma
IL-1 Ra: Interleukine 1 receptor antagonist
IL-1: Interleukine 1
LSQ: Life Situation Questionnaire
LYSA: Lymphoma Study Association
MFI-20: Multidimensional Fatigue Inventory 20-item self-report instrument
NHL: Non-Hodgkin lymphoma
